# The Shoulder Pain and Disability Index demonstrates factor, construct and longitudinal validity

**DOI:** 10.1186/1471-2474-7-12

**Published:** 2006-02-10

**Authors:** Joy C MacDermid, Patty Solomon, Kenneth Prkachin

**Affiliations:** 1 Clinical Research Lab, Hand and Upper Limb Centre, St. Joseph's Health Centre, London, Ontario N6A 4L6, Canada; 2 School of Rehabilitation Science, McMaster University, Hamilton, Ontario L8S 1C7, Canada; 3 Psychology Department, University of Northern British Columbia, Prince George, BC V2N 4Z9, Canada

## Abstract

**Background:**

The Shoulder Pain and Disability Index (SPADI) is a self-report measure developed to evaluate patients with shoulder pathology. While some validation has been conducted, broader analyses are indicated. This study determined aspects of cross-sectional and longitudinal validity of the SPADI.

**Methods:**

Community volunteers (n = 129) who self-identified as having shoulder pain were enrolled. Patients were examined by a physical therapist using a standardized assessment process to insure that their pain was musculoskeletal in nature. This included examination of pain reported during active and passive shoulder motion as reported on a visual analogue pain scale. Patients completed the SPADI, the Coping Strategies Questionnaire (CSQ) and the Sickness Impact Profile (SIP) at a baseline assessment and again 3 and 6 months later. Factor analysis with varimax rotation was used to assess subscale structure. Expectations regarding convergent and divergent subscales of CSQ and SIP were determined a priori and analysed using Pearson correlations. Constructed hypotheses that patients with a specific diagnosis or on pain medication would demonstrate higher SPADI scores were tested. Correlations between the observed changes recorded across different instruments were used to assess longitudinal validity.

**Results:**

The internal consistencies of the SPADI subscales were high (α > 0.92). Factor analysis with varimax rotation indicated that the majority of items fell into 2 factors that represent pain and disability. Two difficult functional items tended to align with pain items. Higher pain and disability was correlated to passive or negative coping strategies, i.e., praying/hoping, catastrophizing on the CSQ. The correlations between subscales of the SPADI and SIP were low with divergent subscales and low to moderate with convergent subscales. Correlations, r > 0.60, were observed between the SPADI and pain reported on a VAS pain scale during active and passive movement. The two constructed validity hypotheses (on diagnosis and use of pain medications) were both supported (p < 0.01). The SPADI demonstrated significant changes over time, but these were poorly correlated to the SIP or CSQ suggesting that these scales measure different parameters.

**Conclusion:**

The SPADI is a valid measure to assess pain and disability in community-based patients reporting shoulder pain due to musculoskeletal pathology.

## Background

The shoulder pain and disability index (SPADI) [1] is a self-report questionnaire developed to measure the pain and disability associated with shoulder pathology. The SPADI consists of 13 items in two subscales: pain (5 items) and disability (8 items); originally items were presented in a visual analogue format. The initial validation work was based on 37 male patients with shoulder pain. High internal consistency (0.86 to 0.95) was observed overall, and moderate test-retest reliability was reported (ICC = 0.65) on a subgroup of 23 patients. Principal components factor analysis with, and without, varimax rotation were conducted, with the former supporting the two subscales currently used; i.e., pain and disability. Validity was established by correlating SPADI total and subscale scores with shoulder range of motion (ROM). The ability to measure clinical change was indicated by high negative correlations between changes in SPADI scores and changes in shoulder ROM [1].

A second group of investigators conducted a larger validation study in 1995 [2]. This study established a numeric (0–10) version of the SPADI, suitability for telephone administration, convergent validity with other health status measures, and responsiveness. Primary care patients (n = 102) with shoulder discomfort were followed for 3 months. Convergent validity was determined by comparing the SPADI with aspects of general health measured by the Health Assessment Questionnaire (HAQ) and the Medical Outcomes Study SF-20 (SF-20). At baseline the visual analogue (VAS) and numeric scaled SPADI were highly concordant (intraclass correlation coefficient = 0.86), and the SPADI correlated substantially with the HAQ (r = 0.61) and the physical functioning (r = -0.50) and pain (r = -0.43) domains of the SF-20. The change in SPADI scores discriminated accurately between subjects who reported being improved versus those who said they were the same or worsened [2].

Support for validity in these initial situations encouraged others to use the SPADI in clinical practice and research. Additional studies were conducted to examine the validity of the SPADI either in isolation or in comparison to other shoulder instruments [3-14] (These are summarized in appended supplementary tables. See Additional file 1). All have provided additional support for the SPADI, although the nature of the subscales has been questioned in subsequent factor analyses [9]. Factor analysis, without varimax rotation in both the original study [1] and in a subsequent one [9] support items loading on a single factor, while varimax rotation supported the 2 subscales currently accepted [1]. This has important implications for scoring and reporting, as well as interpretation. For example, we were using the SPADI in a study examining pain coping, and felt it was important to know whether these subscales separately reflected pain and shoulder-related disability.

The SPADI is only one of many joint-specific self-report forms that focuses on the shoulder. Previous narrative reviews have reflected this spectrum [15]. More recently, a systematic review of shoulder self-report scales was conducted to make definitive conclusions about their methodological properties [16]. It suggested that the construct and responsiveness of the SPADI were good. This review was unable to make definitive conclusions or recommend one instrument over another for any given purpose because of a lack of sufficient methodological studies. It did indicate that the DASH (Disability Arm, Shoulder, Hand) SPADI, ASES (American Shoulder and Elbow Surgeons) were the most studied of 16 identified instruments. However, this review also noted that all instruments require additional methodological evaluation. Subsequent studies have suggested that the SPADI is highly correlated with the DASH and ASES in patients with shoulder arthroplasty [14] and has high responsiveness to detect change following an initial episode of shoulder pain [7] or a spectrum of shoulder conditions [17]. We wished to conduct a study on pain coping styles. This provided an opportunity and need for us to address SPADI validation issues. Firstly, we felt it was important to clarify the subscale (factor) structure of the SPADI in a larger sample size to determine whether subscales scores were valid. Secondly, we felt a broader understanding of the validity and meaning of the scores obtained on the SPADI would be possible by evaluating the impact of different pain coping strategies on SPADI scores. We wish to examine construct validity by comparing SPADI scores to joint irritability and the extent of shoulder pathology. Our final purpose was to supplement the published comparisons of the SPADI with general health status measures (HAQ or SF-20) with a comparison to different health status measure (the SIP) that would address different domains of health.

## Methods

### Subjects

Participants were 129 community volunteers who self-identified in response to newspaper ads or clinic posters recruiting patients having shoulder pain. All participants completed a screening examination that included self-report measures and physical assessment by a physical therapist. Subjects who were unable to read or write in English were excluded from the study. Subjects were participating in a larger study examining behavioural measures of pain.

The study was approved by the McMaster University Research Ethics Board.

### Test Procedures

All patients were examined using a standardized physical examination to insure the pain was attributable to musculoskeletal dysfunction. This assessment included joint irritability, which was assessed by having the patient rate on a VAS pain scale, the extent of pain with active and passive shoulder movement (flexion, abduction, internal, and external rotation). For this study, these scores were averaged.

**Figure 1:**
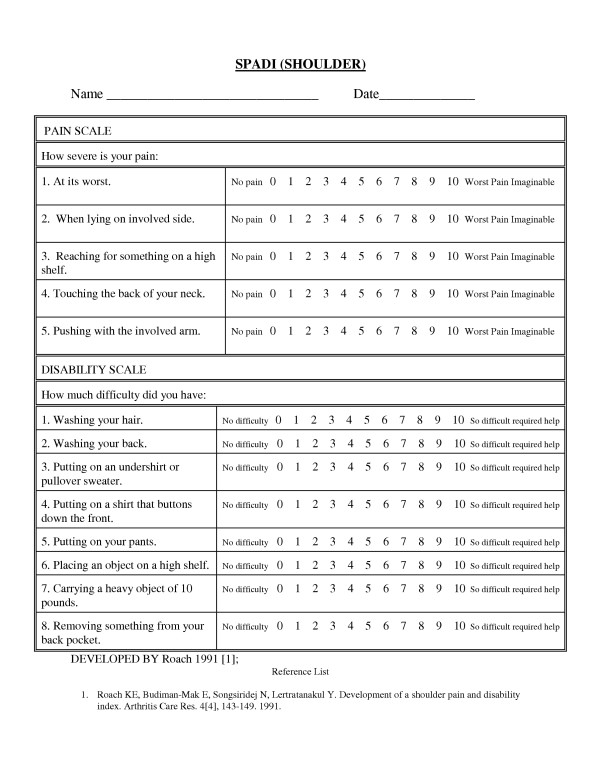
**The Shoulder Pain and Disability Index (SPADI)**. This is a numeric version of the SPADI, used with permission of the developer K Roach.

All subjects completed the numeric version of the SPADI (Figure 1) [1]. The VAS was originally published as a VAS scale, and subsequently validated as numeric scale of 0–10 [2]. The Coping Strategies Questionnaire (CSQ) [18] and a general health measure, the Sickness Impact Profile (SIP) [19-21] were completed on the same occasions. All scales were completed at enrolment and at 2 subsequent follow-up evaluations, 3 and 6 months later.

The CSQ [18] is a 44-item scale with 2 general questions and 7 subscales: Diverting attention, Reinterpreting pain sensation, Coping self statements, Ignoring sensations, Praying/hoping, Catastrophizing, and Increase behavioural act (version obtained from developer). This scale has variations across studies and the form used in this study was obtained directly from the developer.

The SIP [19-21] has 136 items divided across the following domains (number of items in brackets): Sleep and rest (7), Emotional behaviour (9), Body care and movement (23), Home management (10), Mobility (10), Social interaction (20), Ambulation (12), Alertness (10), Communication (9), Work (9), Recreation and past time (8), and Eating (9). In addition, the physical and mental summary scores are computed.

Most subjects had some form of physical therapy for their shoulder problem, i.e., 82% for the total population. See Table 1 for a summary of demographic data. The study neither controlled therapy nor collected details about the type of therapy provided; although none had surgery over the course of the study.

### Analyses

All data analyses were conducted using SPSS 13.0. The distributions of our scores, skewness and kurtosis suggested data were normally distributed. Internal consistency was determined using Cronbach's alpha. Validity was evaluated by conducting three types of validation analyses: factor, construct and longitudinal. Factor analyses with and without varimax rotation were used to evaluate the factor structure of the SPADI. Cross-sectional construct validity analyses were performed separately at each of the 3 time-points. Longitudinal validity was determined across the 3 assessment points by correlating changes observed on different instruments.

Convergent and divergent validity were determined by comparing correlations with SPADI scores across related aspects of pain behaviour (CSQ) and the general health scale (SIP) using Pearson r correlations. SPADI scores were also correlated to joint irritability scores. The clinical significance of correlations is debatable, as a variety of benchmarks have been described. We described the association of different constructs using correlations and rated the effect size of these as defined by Cohen where the effect sizes for correlation coefficients are: r ≈ 0.10 is small effect with negligible practical importance, r ≈ 0.30 is a medium effect with moderate practical importance and r ≈ 0.50 is a large effect of crucial practical importance [22].

Construct validity was evaluated by testing two hypotheses. The first hypothesis was that subjects with diagnosed shoulder problems would have more severe pathology, and therefore more pain and disability than those who complained of shoulder pain, but did not have a specific diagnosis. The second hypothesis was participants who were taking pain medication for their shoulder problem would have higher SPADI scores. These hypotheses were tested using a generalized linear model (ANOVA), which evaluated the changes across the repeated factor (time) and between the two hypothesis-groups (medication or diagnosis hypotheses tested). Finally, longitudinal validity was evaluated by correlating changes on the SPADI to changes in pain subscales on the SIP that were expected to be affected by shoulder pain, i.e., home maintenance and physical health.

## Results

**Figure 2:**
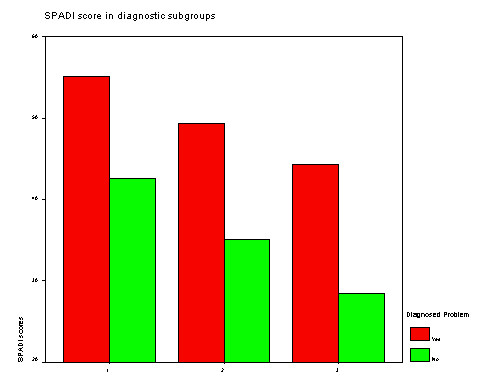
**SPADI scores over time according to diagnostic subgroups**. This graph shows the SPADI score in patients that had a specific diagnosis for their shoulder pain and those who did not. Significant improvements occurred across time in both groups. Patients with a specific diagnosis had higher pain and disability at all 3 time-points (p < 0.001).

**Figure 3:**
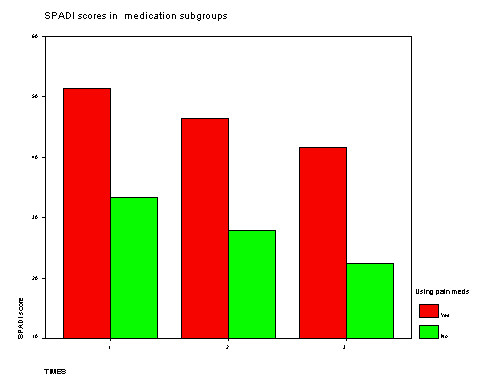
**SPADI scores over time according to pain medication subgroups**. This graph shows the SPADI score in patients that had were using pain medication (at baseline evaluation) as compared to those who were not. Significant improvements in SPADI scores occurred across time in both groups. Patients that were using pain medication had higher pain and disability at all 3 time-points (p < 0.001).

Patient characteristics are in Table 1. The internal consistencies of the total SPADI (α > 0.95), its pain subscale (α > 0.92) and disability subscale (α > 0.93) were very high. Factor analysis with varimax rotation indicated that the majority of items fell into 2 factors that represent pain and disability, respectively, on each of the three assessment occasions. Overall, the factor analyses are consistent with the two subscales: pain and disability. Certain functional items although loading on both factors tended to align more with pain items and one pain item that included a "reaching" component loaded on the pain factor in 1 of 3 time points (See Table 2). The correlations between subscales of the SPADI and CSQ and joint irritability at baseline are in Table 3. The relationship between SPADI and CSQ scores indicated that pain catastrophizing and praying/hoping strategies were associated with medium-sized effects on self-reported pain and disability. Other pain coping strategies had small or non-significant effects. Correlations between the SPADI and joint irritability were more convergent and demonstrated large effects (r > 0.60). Analysis with SIP scores (Table 4) demonstrate that the correlation between the SPADI and divergent subscales of the SIP were low. The relationship with convergent scales was higher, but only reached a moderate level, with the exception of the work subscale where correlations were unexpectedly low. The construct validity hypotheses were both supported (Figure 2 and Figure 3). There were significant differences across time for all patients; but both patients who had diagnosed shoulder problems and those on pain mediation reported higher pain and disability scores on the SPADI at all time points (Figures 2 and 3). In fact, improvement over time in SPADI scores was nearly parallel in both comparisons, indicating similar rates of improvement over time. Although significant improvements in pain and disability occurred over time (p < 0.001), correlations between changes in scores on SPADI subscales and changes on the SIP or CSQ were low and non-significant except for pain catastrophizing.

## Discussion

This study provides additional support for the use of the SPADI as a measure of pain and disability in patients with shoulder pain. Despite the availability of outcome measures specific to upper extremity it has been shown that the rates of utilization of self-report measures are low in clinical practice [23]. Brevity and simplicity are properties that are highly valued by clinicians when considering whether to use an outcome measure in clinical practice [24]. The structure of the SPADI suggests that it would be practical in the clinical setting, particularly the numeric format used in this study (Figure 1), which lessens the scoring burden on the clinician when compared to the visual analogue version. Self-report disability measures are key outcomes in orthopaedic clinical trials. In this case, responsiveness is a prime concern as it determines sample size requirements. There are insufficient data from head-to-head comparisons of available shoulder instruments to identify the most responsive instrument for different shoulder pathologies [16]. This study did not address the validity or responsiveness relative to competing measures, but contributes to current studies providing support for the SPADI as an option in clinical evaluation by providing additional evidence that the SPADI discriminates between subgroups and detects change over time.

Some of the current findings substantiate the results of previous validation. For example, Roach [1] originally reported a two factor solution to factor analysis when varimax rotation was used. However, that study sample was much lower than typically required to conduct factor analyses, which would suggest that there might be some instability in their results. Later, data from a much larger sample [9] suggested that the SPADI contained a single factor, although only unrotated analyses were performed. Our study indicated high internal consistency across SPADI items and an unrotated factor analyses indicated that 57% of variance loaded on a single factor. Varimax rotation has been recommended as it is preferable when inter-item correlations are anticipated [25] such as in the case of pain and disability items. The outcome of this analysis was consistent with the analysis performed in the original validation study [1]. Our results supported the concept of separate pain and disability subscales, although the items on *overhead reaching *and *carrying heavy objects *tended to load with pain in 2/3 of the evaluations. This may reflect that these higher demand activities are most painful for patients with shoulder problems. In one case, a pain item that included a functional component (reaching) loaded with disability items. Others have questioned the extent to which functional items are independent from pain items on the WOMAC's assessment of lower extremity musculoskeletal problems, particularly when items ask about pain during a functional task [26,27]. Our results suggest that some specific functional items might be difficult to separate from pain in musculoskeletal pathologies affecting the shoulder, and that items that include a reference to both pain and function may load on either factor. In situations where factorial analyses are quite different from the subscales described by developers, it might be advisable to use only total scores in clinical or research reporting, particularly if the subscale structure does not hold up across repeated clinical studies. Given that some instability in factorial analyses across studies/conditions might be anticipated, and that most items followed the pain and disability subscale structure described for the SPADI, we would interpret our findings as supporting the existing pain and disability subscales of the SPADI.

Previous studies have compared SPADI scores to shoulder motion and found that patients with better movement had less pain and disability [2]. We have focused on pain during motion as a comparator and demonstrated moderately strong correlation, providing evidence that joint irritability as defined in this study was related to pain and disability reported on the SPADI.

Other studies have correlated the SPADI with instruments measuring function (DASH, SST), pain or general health (SF-36). Our results are in general agreement with others who have reported that upper extremity instruments, including the SPADI are moderately related to physical subscales of the SIP [5,28,29]. Others have suggested that the SIP may exhibit ceiling effects with healthier subjects [30], which might be a consideration given the age and sampling used in our study. We had anticipated that SIP measures of disability on some physical dimensions, like work, might have demonstrated stronger correlations with the SPADI. However, others have reported that the SIP is less correlated with musculoskeletal measures than is the SF-36 [31], suggesting that inherent properties of the SIP may explain the correlations observed. Similarly, we found that while the SPADI detected significant changes at each time point following assessment, these correlated poorly with changes on the SIP. Others have suggested that the SIP is less responsive in musculoskeletal problems than the SF-36 [31,32] or suggested that despite its longer length, responsiveness was no better [33]. Furthermore, we know that even the SF-36 is much less responsive than measures specific to the upper extremity [34]. We interpret these findings as support for our conclusion that the low correlations between SPADI and SIP items observed on longitudinal change reflect a lack of responsiveness on the SIP to changes in health emanating from changes in shoulder status. This reflects an inherent methodological problem when comparing change on more responsive instruments to that occurring on less responsive instruments.

This study adds to previous validation studies in that a novel scale that addresses pain coping behaviour was included. This provides broader support for the SPADI and new information on the relationships between different aspects of pain coping and pain reporting on the SPADI. We were able to determine that negative or passive coping strategies were associated with higher levels of reported disability at each cross-sectional analysis. Previous research has illustrated the importance of pain catastrophizing as a determinant of self-reported pain or disability in other musculoskeletal problems [35-41]. Others have found that changes in pain catastrophizing were associated with clinical improvement in chronic pain patients [42]. Our findings suggest both of these phenomena also occur in shoulder pain. Changes in pain catastrophizing were associated with changes in SPADI scores, whereas other elements of pain coping were not associated with changes in SPADI scores. This may reflect a lack of responsiveness of the instrument(s) or suggest that these other strategies for coping with pain are less likely to change over time.

This study validates the SPADI for usage in patients presenting at the primary care level with shoulder pain. Our study did not determine the specific diagnosis, although a physical therapist confirmed it was of a musculoskeletal nature. Therefore, it was impossible to determine which patients had resolution of their problem, preventing traditional responsiveness analyses. A further limitation is that we did not compare the SPADI to competing shoulder instruments. Despite these limitations, this study indicates that the SPADI detects change over time in excess of that reported on the SIP and that the extent of this change fits with constructed hypotheses. This supports the validity of the SPADI as a measure of shoulder pain and disability. This study also highlights that patients with different responses to pain may report pain and disability differently confirming the importance of serial measurements when assessing the response to interventions and suggesting that further study on this phenomena and how it relates to treatment response is warranted. Given the number of shoulder instruments reported in the literature, head-to-head comparisons determining the ability of different scales to detect treatment responses in different clinical situations are needed.

## Conclusion

This study provides additional support for the validity of the SPADI in clinical evaluation of shoulder pain and disability in that it discriminates between levels of pain and disability in a community-based sample. It provides new information suggesting that negative pain coping strategies are associated with greater self-reported pain and disability. Future studies should focus on comparing competing measures to encourage greater uniformity or comparability across future clinical outcomes studies.

## Competing interests

The author(s) declare that they have no competing interests.

## Authors' contributions

PS and KP formed the original study team that obtained the ethics approval and obtained grant funding for a study on facial recognition of pain. JM consulted on shoulder scales during grant development and worked with PS to develop the research questions around instrument validation. PS coordinated data collection/management. JM conducted statistical analyses and drafted the manuscript. All authors approved the final study protocol, contributed to interpretation of the study results and participated in revisions of the manuscript. All authors read and approved the final manuscript.

## Pre-publication history

The pre-publication history for this paper can be accessed here:



## Supplementary Material

Additional File 1 Supplementary Tables 1–3 On Reliability, Validity and Responsiveness of SPADI reported in previous studies (word file with tables). Click here for file

## Figures and Tables

**Table 1:** Patient Characteristics

Patient Characteristics	Percentage
Gender	49% male, 51% female
Age	44.44 (Range = 19–68)
Affected side	Left 26%, right 42%, both 33%
Education	High school 10%, University 47%, Graduate training 26%
Employment Status	Currently employed 57% Family/unpaid work 2% Student 25% Retired 16%
Shoulder Diagnosis Identified	70%
Using Pain Medications	50%
Dominance	9% left, 91% right
Referral Source	Physiotherapy Clinics 61% Newspaper ads 39%

Baseline Status	Mean (SD)

CSQ Diverting attention	1.72 (1.40)
CSQ Reinterpreting pain sensations	1.06 (1.10)
CSQ Coping self-statements	3.99 (1.22)
CSQ Ignoring sensations	2.57 (1.26)
CSQ Praying/hoping	1.95 (1.58)
CSQ Catastrophizing	1.43 (1.30)
CSQ Increase behavioural act	2.11 (1.41)

SPADI Pain subscale	53.02 (23.28)
SPADI Disability subscale	32.35 (22.84)
SPADI Total	42.82 (21.80)

SIP Sleep and rest	13.50 (16.06)
SIP Emotional behaviour	12.02 (13.25)
SIP Body care and movement	4.20 (7.40)
SIP Home management score	11.90 (15.94)
SIP Mobility score	2.45 (6.71)
SIP Social interaction	7.88 (13.52)
SIP Ambulation	3.57 (8.54)
SIP Alertness behaviour	10.78 (19.51)
SIP Communication	3.56 (6.56)
SIP Work category	16.32 (22.99)
SIP Recreation and past times	19.53 (19.01)
SIP Eating	1.80 (4.32)
SIP Physical dimension	3.70 (6.70)
SIP Psychosocial dimension	8.44 (11.99)
SIP Total	6.07 (8.58)

VAS	3.56 (2.05)

Follow-Up Rates	

3-months	86.0%
6-months	79.8%

**Table 2:** Results of Factor Analysis for SPADI on 3 occasions

	Baseline		3 months		6 months	
Item	1	2	1	2	1	2

Pain at its worst	0.09	**0.76**	**0.84**	0.22	**0.87**	0.20
Pain when lying on involved side	0.24	**0.82**	**0.85**	0.24	**0.88**	0.22
Pain when reaching for something on a high shelf	0.33	**0.81**	**0.85**	0.31	**0.88**	0.30
Pain when touching the back of your neck	0.61	0.52	**0.66**	0.52	**0.68**	0.46
Pain pushing with involved arm	0.27	**0.78**	**0.78**	0.26	**0.74**	0.42
Difficulty washing your hair	0.76	0.40	0.39	0.82	0.44	0.79
Difficulty washing your back	0.69	0.44	0.51	0.72	0.50	0.65
Difficulty putting on an undershirt or pullover sweater	0.66	0.42	0.45	0.76	0.45	0.76
Difficulty putting on a shirt that buttons down the front	0.87	0.05	0.27	0.82	0.22	0.85
Difficulty putting on your pants	0.80	0.16	0.10	0.83	0.14	0.85
Difficulty placing an object on a high shelf	0.49	**0.70**	**0.79**	0.43	**0.72**	0.52
Difficulty carrying a heavy object of 10 pounds	0.47	**0.61**	**0.61**	0.53	0.55	0.56
Difficulty removing something from your back pocket	0.68	0.36	0.40	0.73	0.36	0.79

Extraction Method: Principal Component Analysis. Rotation Method: Varimax with Kaiser Normalization. Rotation converged in 3 iterations. The items falling into the "pain factor" are **bolded **and the items falling into the disability factor are underlined.

**Table 3:** Correlations comparing the SPADI with different pain coping subscales (strategies) and joint irritability

	Diverting attention	Reinterpreting Pain Sensations	Coping Self Statements	Ignoring Sensations	Praying/Hoping	Catastrophizing	Increase Behavioural Act	Joint Irritability VAS
Baseline								

Pain	0.16	0.11	0.10	-0.05	0.32**	0.39**	0.13	0.64**
Disability	0.29**	0.15	0.13	0.05	0.42**	0.41**	0.26**	0.63**
Total	0.24**	0.14	0.12	0.01	0.40**	0.42**	0.21*	0.67**

3-months								

Pain	0.07	-0.04	0.17	0.08	0.24*	0.41**	0.09	
Disability	0.22*	0.15	0.14	0.11	0.33**	0.50**	0.23*	
Total	0.15	0.06	0.17	0.10	0.30**	0.48**	0.16	

6-months								

Pain	0.14	0.07	0.23*	0.17	0.29**	0.41**	0.17	
Disability	0.19	0.09	0.27**	0.21*	0.29**	0.47**	0.15	
Total	0.17	0.08	0.26**	0.20*	0.31**	0.47**	0.17	

** Correlation is significant at the 0.01 level (2-tailed). * Correlation is significant at the 0.05 level (2-tailed). According to Cohen (1987) the effect sizes for correlation coefficients are r ≈ 0.10 is small effect with negligible practical importance, r ≈ 0.30 is a medium effect with moderate practical importance and r ≈ 0.50 is a large effect of crucial practical importance [22].

**Table 4:** Correlations between SPADI subscales and SIP subscales

SPADI score	Communication	Eating	Alertness Behaviour	Mobility	Emotional Behaviour	Ambulation	Sleep & Rest	Body Care and Movement	Social Interaction	Psychosocial dimension	Recreation and Pastime	Home Management	Work	Physical dimension	SIP Total
	Divergent→→→→→→→→→→→→→→→→→→→→→→→→→→Intermediate→→→→→→→→→→→→→→→→→→→→→→→→→→→→→→→→→Convergent														

**Baseline**															
Pain	0.21*	0.22*	0.29**	0.33**	0.34**	0.32**	0.32**	0.42**	0.40**	0.37**	0.36**	0.54**	-0.14	0.43**	0.43**
Disability	0.12	0.22*	0.28**	0.29**	0.30**	0.43**	0.31**	0.58**	0.31**	0.31**	0.36**	0.59**	-0.04	0.55**	0.43**
Total	0.17	0.23**	0.30**	0.32**	0.33**	0.40**	0.33**	0.53**	0.37**	0.36**	0.38**	0.59**	-0.10	0.51**	0.45**
**3-months**															
Pain	0.25**	0.23*	0.33	0.18	0.00	0.31**	0.45**	0.45**	0.46**	0.12	0.36**	0.63	0.16	0.42**	0.19
Disability	0.27**	0.35**	0.37**	0.30**	0.10	0.40**	0.37**	0.62**	0.47**	0.22*	0.35**	0.64**	0.22*	0.58**	0.30**
Total	0.27**	0.31**	0.37**	0.25**	0.05	0.37**	0.44**	0.56**	0.49**	0.18	0.37**	0.67**	0.20*	0.53**	0.26**
**6-months**															
Pain	0.30**	0.08	0.31**	0.14	0.35**	0.28**	0.40**	0.24*	0.30**	0.35**	0.42**	0.50**	0.22*	0.25*	0.34**
Disability	0.35**	0.15	0.48**	0.20*	0.40**	0.35**	0.46**	0.39**	0.45**	0.50**	0.46**	0.51**	0.31**	0.37**	0.49**
Total	0.34**	0.12	0.41**	0.18	0.40**	0.33**	0.46**	0.33**	0.39**	0.44**	0.46**	0.53**	0.27**	0.33**	0.43**

** Correlation is significant at the 0.01 level (2-tailed). * Correlation is significant at the 0.05 level (2-tailed). Columns are order according to hypothesized convergence of constructs with SPADI, i.e., divergent subscales are to the left, the equivocal ones in the middle and convergent construct subscales are on the right. According to Cohen (1987) the effect sizes for correlation coefficients are r ≈ 0.10 is small effect with negligible practical importance, r ≈ 0.30 is a medium effect with moderate practical importance and r ≈ 0.50 is a large effect of crucial practical importance.[22]

**Table 5:** Correlations between changes in health and coping strategies with SPADI

CHANGE SCORES	SPADI PAIN	SPADI DISABILITY	SPADI TOTAL
SPADI Pain		0.66**	0.91**
SPADI Disability	0.66**		0.88**
SPADI Total	0.91**	0.88**	
SIP Home Maintenance	0.10	0.27**	0.20*
SIP Physical Summary	-0.06	0.18	0.06
CSQ Diverting attention	-0.15	-0.15	-0.16
CSQ Reinterpreting Pain Sensations	-0.07	-0.12	-0.09
CSQ Coping Self Statements	0.12	0.16	0.16
CSQ Ignoring Sensations	0.05	-0.03	0.01
CSQ Praying/Hoping	-0.06	-0.02	-0.04
CSQ Catastophizing	0.20*	0.25*	0.25*
CSQ Increase Behavioural Act	0.05	-0.03	0.01

** Correlation is significant at the 0.01 level (2-tailed). * Correlation is significant at the 0.05 level (2-tailed). These correlations suggest that the relationship between changes in overall physical health on the SIP or coping strategies are poorly correlated. Changes in pain catastrophizing were significantly correlated. According to Cohen (1987) the effect sizes for correlation coefficients are r ≈ 0.10 is small effect with negligible practical importance, r ≈ 0.30 is a medium effect with moderate practical importance and r ≈ 0.50 is a large effect of crucial practical importance.[22]
