# Supplementary material for: The Shoulder Pain and Disability Index demonstrates factor, construct and longitudinal validity
Source: BMC Musculoskelet Disord. 2006 Feb 10;7:12. doi: 10.1186/1471-2474-7-12 (PMC1382226; doi:10.1186/1471-2474-7-12)
Supplement: Additional File 1 — Supplementary Tables 1–3 On Reliability, Validity and Responsiveness of SPADI reported in previous studies (word file with tables). [file 1471-2474-7-12-S1.doc]

Table 1. Reliability reported for the SPADI in previous studies

| Study | Population | Type | SPADI Results | | | Comparators | | | | | | |
| --- | --- | --- | --- | --- | --- | --- | --- | --- | --- | --- | --- | --- |
|  |  |  | Pain subscale | Disability subscale | SPADI Total |  |  |  |  |  |  |  |
| Roach et al., 1991[1] | n = 37, Age = 58 (23-76)  M patients with shoulder pain (27 had musculoskeletal origin) | T-R reliability (ICC) (n=23)  I-C (α_c_) (n=33) | 0.64  0.86 | 0.64  0.93 | 0.66  0.95 | None | | | | | | |
| Williams, Holleman & Simel, 1995[2] | n = 102 (98% M), Median age = 60  Patients with shoulder discomfort > 3 months | r between VAS and numeric SPADI (ICC) | -- | -- | 0.86 | None | | | | | | |
| Beaton & Richards, 1998[3], | 99 patients (54% F)  age = 48 (18-77)  Patients with shoulder problems | T-R reliability  (n=55)  ICC | -- | -- | 0.91 | SSRS  0.71 | M-  ASES  0.96 | | SSI  0.97 | SST  0.99 | | SF36  range  =0.86 to 0.98  PCS=  0.96  MCS=0.94 |
| Roddey et al., 2000[4] | n = 192 (58% M); Age = 47 (18-87)  Patients with shoulder pain (46% had shoulder surgery) | I-C (α_c_)  SEM | 0.89  7.82 | 0.95  5.78 | 0.96  4.75 | SST  0.85  11.65 | | | | | | |
| Cook et al., 2001[5] | n = 192 (59% M), Age = 47 (18-72)  Shoulder patients (46% postoperative) | SEM of middle-range scores (50)  Person reliability  (BIGSTEPS calibration) | --  -- | 95% CI of 8.8 units  0.88 | --  -- | UPenn  Function  95% CI of 9.9 units  0.94 | | ASES Function  95% CI of 13.8 units  0.89 | | | SST  95% CI of 36.7 units  0.66 | |
| Bot et al., 2004[6] | Systematic review of literature; method/results of 16 questionnaires  rated | I-C  Reliability  Agreement | --  --  -- | --  --  -- | Doubt-ful  Doubt-  ful  Good | Good: UEFS  Doubtful: SIQ, OSQ, SRQ, SST, ASES  Good: DASH, WOSI,  Doubtful: SIQ, SSRS, SRQ, SST, WOOS, SSI, ASES  Good: SIQ, OSQ, DASH,  Doubtful: RC-QOL, SSRS, SRQ, SST, SSI, ASES | | | | | | |
| Cook et al., 2002[7] | n = 110 (65% M), age = 49.2 (18-78)  Shoulder patients (58% postsurgical) | T-R reliability for postsurgical (ICC) (n=31)  T-R reliability for nonsurgical (ICC) (n=25)  I-C (α_c_) | 0.91  0.70  0.90 | 0.57  0.84  0.94 | 0.91  0.84  -- | UCLA pain and function  0.78,0.89  0.59,0.57  -- | | CMS pain  0.80  0.87  -- | | | ASES pain, function, and total  0.88,0.78  0.91  0.65,0.86  0.84  Function  0.90 | |
| Schmitt & Di Fabio, 2004[8] | n = 211 (50% M), Age = 47.5( 18-88) Musculoskeletal upper extremity problems | T-R reliability for proximal (ICC) (n=35)  SEM for proximal (n=53) | --  -- | --  -- | 0.86  7.75 | DASH  0.91  5.22 | | PRWE  --  -- | | | SF-12-PCS  0.75  4.47 | |
| Cloke et al., 2005[9] | n = 110 (62F)  age = 55 (24-89)  Patients with subacromial impingement | T-R reliability  Weighted κ | -- | -- | range =  0.49 to 0.80 | OSS  Range = 0.12 to 0.79 | | | | | | |
| Ostor et al., 2005[10] | n = 131 (53% M)  age = 57 (18-87)  Patients with shoulder disorders | I-C | α_c_ = 0.81 | α_c_ = 0.90 | -- | None | | | | | | |
| MacDermid, Solomon & Prkachin, submitted | n = 129 (51%F)  age = 44 (19-68)  Patients with shoulder pain | I-C | α_c_ > 0.92 | α_c_ > 0.93 | α_c_ > 0.95 | None | | | | | | |

Legend: α_c_ = Cronbach’s alpha; CI = confidence interval; I-C = internal consistency; ICC = intraclass correlation coefficient; SEM = standard error of measurement; T-R reliability = test-retest reliability

Abbreviations: M- ASES = Modified American Shoulder and Elbow Surgeons Shoulder Index; CMS = Constant-Murley Scale; DASH = Disabilities of the Arm, Shoulder and Hand; F = female; M = male; OSS(Q) = Oxford Shoulder Score (Questionnaire); PRWE = Patient-Rated Wrist Evaluation; RC-QOL = Rotator Cuff Quality of Life Measure; SF-12-PCS = 12-Item Short-Form Health Survey Physical Component Scale; SF-36 MCS = 36-Item Short Form Health Survey Mental Component Score; SF-36 PCS = 36-Item Short Form Health Survey Physical Component Score; SIQ= Shoulder Instability Questionnaire; SPADI = Shoulder Pain and Disability Index; SRQ = Shoulder Rating Questionnaire; SSI = Shoulder Severity Index; SSRS = Subjective Shoulder Rating Scale; SST = Simple Shoulder Test; UCLA = University of California at Los Angeles Shoulder Score; UEFL = Upper Extremity Functional Limitation Scale; UPenn = University of Pennsylvania Shoulder Scale; WOOS = Western Ontario Osteoarthritis of the Shoulder Index; WOSI = Western Ontario Shoulder Instability Index

Table 2. Validity of the SPADI reported in previous studies

| Study | Population | Type | SPADI Results | | | Comparators | | | | | | | | | |
| --- | --- | --- | --- | --- | --- | --- | --- | --- | --- | --- | --- | --- | --- | --- | --- |
|  |  |  | Pain subscale | Disability subscale | SPADI Total |  |  |  |  |  |  |  |  |  |  |
| Roach et al., 1991[1] | n = 37; age = 58 (23-76)  M patients with shoulder pain (27 musculoskeletal origin) | Construct  Factor analysis without rotation  Factor analysis with rotation  Criterion using ROM | --  --  r = -0.54 to -0.80 | --  --  r = -0.52 to -0.77 | 1 factor  2 factors  r = -0.55 to -0.80 | None | | | | | | | | | |
| Williams, Holleman & Simel, 1995[2] | n = 102 (98% M); Median age: 60  Patients with shoulder discomfort > 3 months | Construct  r with HAQ  r with physical and pain functioning of SF-20 | --  -- | --  -- | r = 0.61  r = -0.50,  r = -0.43 | None | | | | | | | | | |
| Beaton et al., 1996[11] | n= 90 (55% M) age = 48 (18-77)  Shoulder patients (61% impingement syndrome) | Construct  r with SST  r with SSRS  r with M-ASES  r with SSI  r with acute SF-36 physical fun.  r with acute SF-36 pain  r with acute SF-36 overall  r with elevation of shoulder | --  --  --  --  --  --  --  -- | --  --  --  --  --  --  --  -- | r_s_ = 0.74  r_s_ = 0.50  r_s_ = 0.77  r_s_ = 0.79  r_s_ = 0.58  r_s_ = 0.64  r_s_ = 0.67  r_s_ = 0.07 | SST  --  r_s_=0.47  r_s_=0.73  r_s_=0.80  r_s_=0.58  r_s_=0.62  r_s_=0.60  r_s_=0.30 | | SSRS  r_s_=0.47  --  r_s_=0.50  r_s_=0.48  r_s_=0.12  r_s_=0.40  r_s_=0.32  r_s_=0.13 | | | M-ASES  r_s_=0.73  r_s_=0.50  --  r_s_=0.79  r_s_=0.60  r_s_=0.58  r_s_=0.67  r_s_=0.45 | | | SSI  r_s_=0.80  r_s_=0.48  r_s_=0.79  --  r_s_=0.59  r_s_=0.65  r_s_=0.72  r_s_=0.26 | |
| Heald, Riddle & Lamb, 1997[12] | n = 94 (59 M)  age = 44.8 (19-82)  Shoulder patients | Construct  r with SIP total, body care and movement, home management scores  r with unrelated SIP scores | r_s_ = 0.44 to 0.51  r_s_ < 0.50 | r_s_ = 0.48 to 0.54  r_s_ < 0.50 | r_s_ = 0.49 to 0.57  r_s_ < 0.50 | None | | | | | | | | | |
| MacDermid et al., 1999[13] | n = 34; age = 55  Shoulder patients | Construct  r with pain intensity measures from movement diagrams  r with pain onset measures from movement diagrams  r with ROM | r = 0.58 to 0.72  r = -0.40 to -0.69  -- | r = 0.56  r = -0.56  r = -0.40 | --  --  -- | None | | | | | | | | | |
| Roddey et al., 2000[4] | n = 192 (58% M), age = 47 (18-87)  Patients with shoulder pain (46% shoulder surgery) | Construct  Factor Analysis  Convergent  r with SPADI pain  r with SST  r with UCLA pain  r with UCLA function | --  --  r_s_ = -0.69  r_s_ = -0.63  r_s_ = -0.61 | --  r_s_ = 0.77  r_s_ = -0.80  r_s_ = -0.54  r_s_ = -0.64 | 1 factor explained 68.4% of total variance  --  --  --  -- | SST  --  --  r_s_ = 0.48  r_s_ = 0.60 | | | UCLA pain  --  r_s_ = 0.48  --  r_s_ = 0.52 | | | | UCLA function  --  r_s_ = 0.60  r_s_ = 0.52  -- | | |
| Beaton et al., 2001[14] | n = 200 (113 F ), age = 42  Shoulder, wrist/hand patients | Construct  r with DASH (whole cohort)  r with DASH (shoulder patients, n=138) | r = 0.82  r = 0.79 | r = 0.88  r = 0.85 | --  -- | None | | | | | | | | | |
| Angst et al., 2004[15] | n = 43 (33F)  age=65.1 (31-87)  Patients who underwent shoulder arthroplasty | Construct  r with SF-36 PCS  r with SF-36 MCS  r with DASH  r with ASES  r with CS | --  --  --  --  -- | --  --  --  --  -- | r_s_ = 0.63  r_s_ = 0.08  r_s_ = 0.93  r_s_ = 0.81  r_s_ = 0.82 | SF-36  MCS=  0.16  PCS=  0.16  PCS=  0.67  MCS=  0.06  PCS=  0.64  MCS=  0.24  PCS=  0.45  MCS=  0.02 | | DASH  r_s_=0.67  r_s_=0.06  --  r_s_=0.79  r_s_=0.82 | | | ASES  r_s_=0.64  r_s_=0.24  r_s_=0.79  --  r_s_=0.71 | | | CS  r_s_=0.45  r_s_=0.02  r_s_=0.82  r_s_=0.71  -- | |
| Bot et al., 2004[6] | Systematic review of literature; method/results of 16 questionnaires  rated | Content  Construct | --  -- | --  -- | Poor  Good | Good: SDQ-UK, SIQ, OSQ, RC-QOL, DASH, WOSI, SRQ, SST, WOOS  Doubtful: SDQ-NL  Poor: UEFS, UEFL, ASES  Good: SDQ-UK, SIQ, OSQ, SDQ-NL, RC-QOL, DASH, WOSI, SST, SSI, ASES, UEFL  Doubtful: SSRS, SRQ, WOOS, UEFS | | | | | | | | | |
| Paul et al., 2004[16] | n = 180 (50% M); age = 53.5(19-85)  Patients with new episode of shoulder pain | Construct  r with SDQ-NL  r with SDQ-UK  r with SRQ  r with shoulder ROM  r with EQ 5  r with EQ TS  r with pain VAS  r with difficulty VAS | --  --  --  --  --  --  --  -- | --  --  --  --  --  --  --  -- | r_s_ = 0.33  r_s_ = 0.57  r_s_ = 0.83  r_s_ = -0.09 to -0.25  r_s_ = -0.47  r_s_ = -0.29  r_s_ = 0.66  r_s_ = 0.62 | SDQ-NL  --  r_s_ = 0.52  r_s_ = 0.43  r_s_ = -0.21 to -0.44  r_s_= -.43  r_s_ = -.45  r_s_ = 0.48  r_s_ = 0.47 | | | SDQ-UK  r_s_ = 0.52  --  r_s_ = 0.72  r_s_ = -0.02 to -0.34  r_s_ = -.68  r_s_ = -.45  r_s_ = 0.50  r_s_ = 0.41 | | | | SRQ  r_s_ = 0.43  r_s_ = 0.72  --  r_s_ = -0.23 to -0.44  r_s_ = -.50  r_s_ = -.43  r_s_ = 0.62  r_s_ = 0.60 | | |
| Placzek et al., 2004[17] | n = 70 (56% M)  age = 54.6 (22-87)  Patients with shoulder problems | Construct  r with SPADI pain  r with SPADI function  r with SPADI total  r with ASES pain, function  r with ASES total  r with Constant pain, function  r with Constant total  r with UCLA pain, function  r with UCLA total  r with SST  r with Wolfgang pain, function  r with Wolfgang total | --  r = 0.71  r = 0.92  r = -0.57  --  r = -0.33  --  r = -0.43  --  --  r = -0.48  -- | r = 0.71  --  r = 0.93  r = -0.67  --  r = -0.26  --  r = -0.45  --  --  r = -0.31  -- | r = 0.92  r = 0.93  --  --  r = -0.77  --  r = -0.56  --  r = -0.68  r = -0.69  --  r = -0.58 | AS-  ES  P:  -0.57  F:  -0.67  T:  -0.77  r = 0.58  P:  0.43  F:  0.82  P:  0.48  F:  0.35  T: 0.50  P:  0.49  F:  0.45  T: 0.66  T: 0.60  P:  0.37  F:  0.49  T: 0.52 | Con-stant  P:  -0.33  F:  -0.26  T:  -0.56  P:  0.48  F:  0.35  T:  0.50  r =  0.10  P:  0.55  F:  0.44  P:  0.31  F:  0.20  T: 0.59  T: 0.65  P:  0.40  F:  0.31  T: 0.68 | | | UC-LA  P:  -0.43  F:  -0.45  T:  -0.68  P:  0.49  F:  0.45  T:  0.66  P:  0.31  F:  0.20  T:  0.59  r =  0.16  P: 0.67  F:  0.73  T: 0.56  P:  0.37  F:  0.43  T: 0.62 | | Wolfgang  P:  -0.48  F:  -0.31  T:  -0.58  P:  0.37  F:  0.49  T:  0.52  P:  0.40  F:  0.31  T:  0.68  P:  0.37  F:  0.43  T: 0.62  T: 0.68  r = 0.35  P:  0.60  F:  0.70 | | | SST  --  --  r =  -0.69  --  r =  0.60  --  r =  0.65  --  r =  0.56  --  --  r =  0.68 |
| Cloke et al., 2005[9] | n = 110 (62F)  age = 55 (24-89)  Patients with subacromial impingement | Construct  r with OSS  r with SF-36 Pain  r with SF-36 PF  r with SF-36 Total | --  --  --  -- | --  --  --  -- | r = 0.85  r = 0.65  r = -0.50  r = -0.26 | OSS  --  r = 0.69  r = -0.57  r = -0.37 | | | | | | | | | |
| Roddey et al., 2005[4] | n = 108  Patients who underwent arthroscopic repairs of rotator cuff tears | Construct  r with strength estimates  r with ROM | r = -0.43  r = -0.46 | r = -0.53  r = -0.49 | r = -0.49  r = -0.48 | UPenn pain  r = 0.29  -- | | | UPenn function  r = 0.48  r = 0.59 | | | | UPenn total  r = 0.44  r = 0.50 | | |
| MacDermid, Solomon & Prkachin, submitted | n = 129 (51%F)  age = 44 (19-68)  Patients with shoulder pain | Convergent & Divergent  Baseline:  r with CSQ subscales  r with SIP subscales  r with VAS  3-months:  r with CSQ subscales  r with SIP subscales  6-months:  r with CSQ subscales  r with SIP subscales  Construct  Patients with diagnosis have higher scores  Patients taking meds have higher scores | r=-0.05 to  0.39  r=-0.14 to 0.54  r = 0.64  r=-0.04 to  0.41  --  r=0.07 to 0.41  r=0.08 to 0.50  p < 0.001  p < 0.001 | r=0.05 to 0.42  r=-0.04 to 0.59  r = 0.63  r=0.11 to  0.50  r=0.10 to 0.64  r=0.09 to 0.47  r=0.15 to 0.51  p < 0.001  p < 0.001 | r=0.01 to 0.42  r=-0.10 to 0.59  r = 0.67  r=0.06 to  0.48  r=0.05 to 0.67  r=0.08 to 0.47  r=0.12 to 0.53  p < 0.001  p < 0.001 | None | | | | | | | | | |

Legend: r = Pearson correlation coefficient; r_s_ = Spearman’s correlation coefficient; M = male, F = female

Abbreviations: M-ASES = Modified American Shoulder and Elbow Surgeons; CS = Constant Murley Scale; DASH = Disabilities of the Arm, Shoulder and Hand; EQ = EuroQol; OSS(Q) = Oxford Shoulder Score(Questionnaire); RC-QOL = Rotator Cuff Quality of Life Measure; ROM = range of motion; SDQ-NL = Dutch Shoulder Disability Questionnaire; SDQ-UK = United Kingdom Shoulder Disability Questionnaire; SF-12 PCS = 12-Item Short Form Health Survey Physical Component Score; SF-36 MCS = 36-Item Short Form Health Survey Mental Component Score; SF-36 PCS = 36-Item Short Form Physical Component Score; SIP = Sickness Impact Profile; SIQ = Shoulder Instability Questionnaire; SPADI = Shoulder Pain and Disability Index; SRQ = Shoulder Rating Questionnaire; SSI = Shoulder Severity Index; SSRS = Subjective Shoulder Rating Scale; SST = Simple Shoulder Test; UCLA = University of California at Los Angeles Shoulder Score; UPenn = University of Pennsylvania Shoulder Scale; UEFL = Upper Extremity Functional Limitation Scale; UEFS = Upper Extremity Function Scale; VAS = Visual Analogue Scale; WOOS = Western Ontario Osteoarthritis of the Shoulder Index; WOSI = Western Ontario Shoulder Instability Index;

Table 3. Responsiveness to Change (or Longitudinal validation) reported for the SPADI in previous studies

| Study | Population | Type | SPADI Results | | | Comparators | | | | | | | |
| --- | --- | --- | --- | --- | --- | --- | --- | --- | --- | --- | --- | --- | --- |
|  |  |  | Pain subscale | Disability subscale | SPADI Total |  |  |  |  |  |  |  |  |
| Roach et al., 1991[1] | n = 37 M; shoulder pain | Longitudinal validity (n=30)  r with ROM | r = -0.52 to -0.70 | r = -0.50 to -0.63 | r = -0.52 to -0.70 | None | | | | | | | |
| Williams, Holleman & Simel, 1995[2] | n = 102 (98% M); Median age = 60; shoulder discomfort > 3 months | Longitudinal validity r with change in overall status at 2, 4, 12 weeks  ROC (AUC) | --  -- | --  -- | r_s_ = 0.73 to 0.79  0.91 | None | | | | | | | |
| Heald, Riddle & Lamb, 1997[12] | n = 94 (59 M);  Age = 44.8 (19-82); shoulder pain | SRM | 1.54 | 1.04 | 1.38 | SIP Physical  0.15 | | SIP Psycho-social  0.47 | | | | SIP total  0.79 | |
| Beaton & Richards, 1998[3] | 99 patients (54% F)  age = 48 (18-77)  Patients with shoulder problems | SRM (n=33) |  |  | 1.23 | SSRS  0.65 | M-  ASES  0.93 | | SSI  1.05 | | SST  0.87 | | SF36  range  =0.10 to 0.91  PCS=0.55  MCS=0.08 |
| Beaton et al., 2001[14] | N = 200 (113 F), Age = 42;  Shoulder, wrist/hand patients | SRM of all patients  SRM of shoulder patients | --  -- | 0.62 - 0.86  0.71 - 1.13 | --  -- | DASH  0.78-1.20  0.81-1.44 | | | | | | | |
| Bot et al., 2004[18] | Systematic review of literature; method/results of 16 questionnaires  rated | Responsiveness | -- | -- | Good | Good: SIQ, OSQ, SDQ-NL, DASH  Doubtful = WOSI, SSRS, SRQ, SST, WOOS, SSI, UEFS, ASES | | | | | | | |
| Buchbinder et al., 2004[19] | n = 50 (24 prednisolene, 26 placebo)  Age ≥ 18 years  Pain and stiffness in one shoulder for ≥ 3 weeks  Passive motion restricted by >30˚ in 2 or more plans | SRM of active  SRM of placebo  ES of active  ES of placebo | --  --  --  -- | --  --  --  -- | 1.11-1.42  0.64-1.83  2.06-2.28  0.77-2.76 | Croft  0.74-1.43  0.20-1.29  0.98-1.21  0.17-1.51 | | DASH  0.67-1.51  0.10-0.95  0.81-1.18  0.11-1.30 | | | | HAQ  0.75-0.88  0.25-1.17  0.56-0.78  0.11-0.78 | |
| Paul et al., 2004[16] | n = 180 (50% M); Age= 53.5 (19-85); new episode of shoulder pain | ES  SRM  RR  r with self-related change  ROC (AUC) | --  --  --  --  -- | --  --  --  --  -- | 1.52  1.17  1.67  r_s_ = 0.61  0.87 | SDQ-NL  1.56  0.95  1.73  r_s_ = 0.58  0.77 | | SDQ-UK  0.91  0.78  1.39  r_s_ = 0.54  0.77 | | | | SRQ  1.64  1.23  2.76  r_s_ = 0.68  0.85 | |
| Schmitt & Di Fabio, 2004[8] | N = 211 (50% M); Age = 47.5 (18-88); musculoskeletal upper extremity problems; divided into proximal and distal problems; proximal (n=95) data included here | ES of proximal  SRM of proximal  RR of proximal  r with Global Change for proximal  Reliable change proportion  MID proportion | --  --  --  --  --  -- | --  --  --  --  --  -- | 1.21  1.08  1.53  r_s_ = 0.64  0.48  0.56 | DASH  1.06  1.08  1.78  r_s_ = 0.66  0.50  0.58 | | | | SF-12-PCS  1.20  1.07  1.40  r_s_ = 0.55  0.29  0.48 | | | |
| Cloke et al., 2005[9] | n = 110 (62F)  age = 55 (24-89)  Patients with subacromial impingement | Effect Size  1-2 intervals  2-3 intervals  3-4 intervals  1-4 intervals | --  --  --  -- | --  --  --  -- | -0.23  -0.40  -0.19  -0.98 | OSS  -0.24  -0.37  -0.25  -0.96 | | | | SF-36  Pain: -0.17  Phys Func: 0.12  Total: 0.08  Pain: -0.21  Phys Func: 0.02  Total: -0.03  Pain: -0.27  Phys Func: 0.31  Total: 0.18  Pain: -0.76  Phys Func: 0.34  Total: 0.08 | | | |
| MacDermid, Solomon & Prkachin, submitted | n = 129 (51%F)  age = 44 (19-68)  Patients with shoulder pain | Change scores  r with SPADI pain  r with SPADI disability  r with SPADI total  r with SIP pain subscales  r with CSQ subscales | --  r = 0.66  r = 0.91  r = -0.06, 0.10  r = -0.15 to 0.20 | r = 0.66  --  r= 0.88  r = 0.18, 0.27  r= -0.15 to 0.25 | r = 0.91  r = 0.88  --  r = 0.06, 0.20  r = -0.16 to 0.25 | None | | | | | | | |

Legend: AUC = area under the curve; ES = effect size; MID = minimal important difference; r = Pearson correlation coefficient; r_s_ = Spearman’s correlation coefficient; ROC = Receiver Operating Characteristic; RR = Guyatt’s Responsiveness Ratio; SRM = standardized response mean; M = male, F = female

Abbreviations: ASES = American Shoulder and Elbow Surgeons Standardized Shoulder Assessment Form; Croft = Croft Shoulder Questionnaire; CSQ = Coping Strategies Questionnaire; DASH = Disabilities of the Arm, Shoulder and Hand; F = function; HAQ = Health Assessment Questionnaire; OSS(Q) = Oxford Shoulder Score (Questionnaire); PRWE = Patient-Rated Wrist Evaluation; ROM = range of motion; SDQ-NL = Dutch Shoulder Disability Questionnaire; SDQ-UK = United Kingdom Shoulder Disability Questionnaire; SF-12-PCS = 12-Item Short-Form Health Survey Physical Component Scale; SF-36 MCS = 36-Item Short Form Health Survey Mental Component Score; SF-36 PCS = 36-Item Short Form Health Survey Physical Component Score; SIP = Sickness Impact Profile; SIQ = Shoulder Instability Questionnaire; SPADI = Shoulder Pain and Disability Index; SRQ = Shoulder Rating Questionnaire; SSI = Shoulder Severity Index; SSRS = Subjective Shoulder Rating Scale; SST = Simple Shoulder Test; T = total; UEFS= Upper Extremity Function Scale; WOOS = Western Ontario Osteoarthritis of the Shoulder Index; WOSI = Western Ontario Shoulder Instability Index;

Reference List

1. Roach KE, Budiman-Mak E, Songsiridej N, Lertratanakul Y: Development of a shoulder pain and disability index. *Arthritis Care Res* 1991, 4: 143-149.

2. Williams JW, Jr., Holleman DR, Jr., Simel DL: Measuring shoulder function with the Shoulder Pain and Disability Index. *J Rheumatol* 1995, 22: 727-732.

3. Beaton D, Richards RR: Assessing the reliability and responsiveness of 5 shoulder questionnaires. *J Shoulder Elbow Surg* 1998, 7: 565-572.

4. Roddey TS, Olson SL, Cook KF, Gartsman GM, Hanten W: Comparison of the University of California-Los Angeles Shoulder Scale and the Simple Shoulder Test with the shoulder pain and disability index: single-administration reliability and validity. *Phys Ther* 2000, 80: 759-768.

5. Cook KF, Gartsman GM, Roddey TS, Olson SL: The measurement level and trait-specific reliability of 4 scales of shoulder functioning: an empiric investigation. *Archives of Physical Medicine & Rehabilitation, 82(11):1558-65, 2001 Nov (64 ref)* 2001, 82: 1558-1565.

6. Bot SD, Terwee CB, van der Windt DA, Bouter LM, Dekker J, de Vet HC: Clinimetric evaluation of shoulder disability questionnaires: a systematic review of the literature. *Ann Rheum Dis* 2004, 63: 335-341.

7. Cook KF, Roddey TS, Olson SL, Gartsman GM, Valenzuela FF, Hanten WP: Reliability by surgical status of self-reported outcomes in patients who have shoulder pathologies. *J Orthop Sports Phys Ther* 2002, 32: 336-346.

8. Schmitt JS, Di Fabio RP: Reliable change and minimum important difference (MID) proportions facilitated group responsiveness comparisons using individual threshold criteria. *J Clin Epidemiol* 2004, 57: 1008-1018.

9. Cloke DJ, Lynn SE, Watson H, Steen IN, Purdy S, Williams JR: A comparison of functional, patient-based scores in subacromial impingement. *J Shoulder Elbow Surg* 2005, 14: 380-384.

10. Ostor AJ, Richards CA, Prevost AT, Speed CA, Hazleman BL: Diagnosis and relation to general health of shoulder disorders presenting to primary care. *Rheumatology (Oxford)* 2005.

11. Beaton DE, Richards RR: Measuring function of the shoulder. A cross-sectional comparison of five questionnaires. *J Bone Joint Surg Am* 1996, 78: 882-890.

12. Heald SL, Riddle DL, Lamb RL: The shoulder pain and disability index: the construct validity and responsiveness of a region-specific disability measure. *Phys Ther* 1997, 77: 1079-1089.

13. MacDermid JC, Chesworth BM, Patterson S, Roth JH: Validity of pain and motion indicators recorded on a movement diagram of shoulder lateral rotation. *Australian Journal of Physiotherapy* 1999, 45: 269-277.

14. Beaton DE, Katz JN, Fossel AH, Wright JG, Tarasuk V, Bombardier C: Measuring the whole or the parts? Validity, reliability, and responsiveness of the Disabilities of the Arm, Shoulder and Hand outcome measure in different regions of the upper extremity. *J Hand Ther* 2001, 14: 128-146.

15. Angst F, Pap G, Mannion AF, Herren DB, Aeschlimann A, Schwyzer HK *et al*.: Comprehensive assessment of clinical outcome and quality of life after total shoulder arthroplasty: usefulness and validity of subjective outcome measures. *Arthritis Rheum* 2004, 51: 819-828.

16. Paul A, Lewis M, Shadforth MF, Croft PR, van der Windt DA, Hay EM: A comparison of four shoulder-specific questionnaires in primary care. *Ann Rheum Dis* 2004, 63: 1293-1299.

17. Placzek JD, Lukens SC, Badalanmenti S, Roubal PJ, Freeman DC, Walleman KM *et al*.: Shoulder outcome measures: a comparison of 6 functional tests. *American Journal of Sports Medicine* 2004 Jul-Aug; 32: 1270-1277.

18. Beaton D, Richards RR: Assessing the reliability and responsiveness of 5 shoulder questionnaires. *J Shoulder Elbow Surg* 1998, 7: 565-572.

19. Buchbinder R, Green S, Forbes A, Hall S, Lawler G: Arthrographic joint distension with saline and steroid improves function and reduces pain in patients with painful stiff shoulder: results of a randomised, double blind, placebo controlled trial. *Ann Rheum Dis* 2004, 63: 302-309.
